# Real-world experience with filgotinib for rheumatoid arthritis in Germany: A retrospective chart review

**DOI:** 10.1007/s00393-024-01506-x

**Published:** 2024-04-30

**Authors:** Olaf Schultz, Christoph Fiehn, Christian Kneitz, Nils Picker, Daniel Kromer, Monia Zignani, Francesco De Leonardis, Hans-Dieter Orzechowski, Margot Gurrath, Klaus Krüger

**Affiliations:** 1 ACURA Kliniken Baden-Baden/Rheumazentrum, Rotenbachtalstraße 5, 76530 Baden-Baden, Germany; 2 Praxis for Rheumatology and Clinical Immunology, Medical Center Baden-Baden, Baden-Baden, Germany; 3 Rheumatologische Schwerpunktpraxis Schwerin, Schwerin, Germany; 4 Ingress-Health HWM GmbH—A Cytel Company, Wismar, Germany; 5 Ingress-Health HWM GmbH—A Cytel Company, Berlin, Germany; 6 Galapagos GmbH, Basel, Switzerland; 7 Galapagos Biopharma Germany GmbH, Munich, Germany; 8 Rheumatologisches Praxiszentrum St. Bonifatius, Munich, Germany

**Keywords:** Janus kinase 1 inhibitors, Clinical trials, Retrospective studies, Rheumatic, Therapeutic rationale, Januskinase-1-Inhibitoren, Beobachtungsstudien, Basistherapie, Therapeutische Grundlage

## Abstract

**Background:**

Real-world data for filgotinib, a Janus kinase (JAK)1 inhibitor, are limited in patients with rheumatoid arthritis (RA).

**Objectives:**

To explore real-world filgotinib use in patients with RA in Germany.

**Materials and methods:**

This retrospective chart review included patients aged ≥ 18 years with confirmed moderate to severe RA who initiated filgotinib before December 1, 2021, with ≥ 6 months of medical records available prior to filgotinib initiation or after initial diagnosis. Patient characteristics, prior treatments, reasons for initiating/discontinuing filgotinib, disease activity, dose adjustments and concomitant treatments were recorded.

**Results:**

In total, 301 patients from 20 German rheumatology outpatient units were included. One-third were aged ≥ 65 years and almost half had ≥ 1 cardiovascular (CV) risk factor. Most patients initiated filgotinib as monotherapy (83.7%; 12.7% of whom with glucocorticoids) and at the 200 mg dose (84.7%); higher proportions of those initiating the 100 versus 200 mg dose were aged ≥ 65 years and had renal impairment or ≥ 1 CV risk factor. Oral administration (78.4%), fast onset of action (66.8%) and administration as monotherapy (65.4%) were the most common reasons for initiating filgotinib. At 12 months, 41 (18.4%) patients had discontinued filgotinib, most commonly due to lack of effectiveness. After 6‑months of follow-up, 36.8% of patients had achieved Clinical Disease Activity Index (CDAI) remission and 45.6% had achieved CDAI low disease activity.

**Conclusions:**

In clinical practice in Germany, reasons for initiating filgotinib in patients with RA were related to dosing flexibility and general JAK inhibitor attributes. Filgotinib was used predominantly as monotherapy and was effective and generally well tolerated; however, longer-term data in larger, prospective cohorts are needed.

**Supplementary Information:**

The online version of this article (10.1007/s00393-024-01506-x) contains tables S1–S4 and figures S1–S3.

## Abstract (de)

**Hintergrund:**

Es existieren nur begrenzt Real-World-Daten zur Anwendung des Januskinase(JAK)-1-Inhibitors Filgotinib (FIL) bei Patienten mit rheumatoider Arthritis (RA).

**Zielsetzung:**

Untersuchung zur Real-World-Anwendung von FIL bei Patienten mit RA in Deutschland (DE).

**Material und Methoden:**

In das retrospektive Chart*-*Review eingeschlossen waren Patienten ≥ 18 Jahre mit bestätigter moderater bis schwerer RA, mit Beginn von FIL vor dem 1. Dezember 2021 und Daten von ≥ 6 Monaten vor FIL-Initiierung oder nach Erstdiagnose. Neben Patientencharakteristika wurden Vortherapien, Gründe für Initiierung/Absetzen von FIL, Krankheitsaktivität, Dosisanpassungen und Begleittherapien erfasst.

**Ergebnisse:**

Einbezogen wurden 301 Patienten aus 20 rheumatologischen Praxen in DE, ein Drittel ≥ 65 Jahre und nahezu die Hälfte mit ≥ 1 kardiovaskulären (CV) Risikofaktor. FIL wurde hauptsächlich als Monotherapie (83,7 %; 12,7 % davon mit Glukokortikoiden) in der 200-mg-Dosierung (84,7 %) begonnen. Patienten mit 100 mg FIL waren häufiger ≥ 65 Jahre alt und wiesen eine Niereninsuffizienz oder ≥ 1 CV-Risikofaktor auf. Häufigste Gründe für FIL waren orale Gabe (78,4 %), schneller Wirkeintritt (66,8 %) und Monotherapie (65,4 %). Nach 12 Monaten hatten 41 Patienten (18,4 %) FIL abgesetzt, hauptsächlich wegen unzureichender Wirksamkeit. Über 6 Monate erreichten 36,8 % der Patienten eine CDAI-Remission (Clinical Disease Activity Index) und 45,6 % eine CDAI-LDA („low disease activity“, geringe Krankheitsaktivität).

**Schlussfolgerungen:**

Gründe für die Therapie mit FIL bei RA in DE waren Dosisflexibilität und allgemeine JAK-Inhibitor-Eigenschaften. FIL wurde hauptsächlich als Monotherapie eingesetzt, war wirksam und i. Allg. gut verträglich. Prospektive Langzeitdaten aus größeren Kohorten sind jedoch noch erforderlich.

## Introduction

The recommended first-line treatment for rheumatoid arthritis (RA) is a conventional synthetic disease-modifying antirheumatic drug (csDMARD), typically methotrexate (MTX), potentially combined with short-term glucocorticoids (GCs) [1–3]. If a patient experiences an inadequate response to MTX and has poor prognostic factors, it is recommended to add an advanced therapy, e.g., a biologic DMARD (bDMARD) or, based on individual risk–benefit assessment and shared decision-making, a Janus kinase (JAK) inhibitor [1–3] to achieve sustained remission or low disease activity (LDA) based on a treat-to-target approach [3, 4].

Filgotinib is a JAK 1-preferential inhibitor that can be used as monotherapy or in combination with MTX in patients with moderate to severe active RA who have an inadequate response or intolerance to one or more DMARDs [5]. Several phase 3 randomized controlled trials (RCTs) have demonstrated efficacy and favorable safety of filgotinib in RA [6–9]; however, real-world data are limited.

The aim of this study was to explore real-world experience with filgotinib in patients with RA in Germany.

## Materials and methods

### Study design and patients

This was a multicenter, retrospective chart review carried out in rheumatology practices in Germany. Patients were eligible if they were ≥ 18 years of age, had a confirmed diagnosis of moderate to severe RA, initiated filgotinib between October 15, 2020 (when it became available in Germany) and December 1, 2021, and had medical records available for ≥ 6 months prior to filgotinib initiation or for ≥ 6 months after initial diagnosis. Patients were excluded if they had participated in an interventional study within 6 months of filgotinib initiation or if they were participating in other observational studies sponsored by Galapagos NV.

The recruitment target was 300 patients with RA treated with filgotinib in Germany across 20 sites. No calculations of sample size were performed as the study sample was determined by the number of eligible patients in the German sites who agreed to participate in the study. Study investigators identified up to 25 patients per site and extracted patient-level data from medical records.

This study was conducted in accordance with the ethical principles of the current Declaration of Helsinki, is consistent with applicable regulatory requirements, and was approved by the Ethics Committee of the Faculty of Medicine at the University of Rostock (reference number A 2022–0009). The protocol was approved by members of the scientific steering committee, including the study sponsor, the research organization conducting the analysis, independent rheumatologists, and one author. Explicit informed consent to access electronic medical record data was not required for this study, as the patients’ privacy was guaranteed, and the data documented by study sites were completely anonymized to the research organization and the study sponsor.

### Objectives and assessments

The primary objective of this study was to evaluate the rationale for initiating filgotinib in patients with moderate to severe RA in Germany. Secondary objectives were to describe the characteristics of patients who initiated filgotinib, concomitant use of GCs and/or MTX and prior treatment with DMARDs. Disease activity and treatment adjustments were included as exploratory objectives.

Data collection using predefined electronic case report forms via an electronic data capture system was completed on May 2, 2022. After data validation, sites were contacted to resolve any data gaps or implausible data that were identified. Data collected included baseline demographics and disease characteristics, serologic status (rheumatoid factor [RF] and anti-cyclic citrullinated peptide antibodies [ACPAs]), prior herpes zoster infection, comorbidities and cardiovascular (CV) risk factors, prior treatments received, and the reasons for initiating filgotinib based on preset response categories. Reasons for discontinuation of filgotinib were evaluated. Disease activity was assessed and classified using Clinical Disease Activity Index (CDAI; remission: ≤ 2.8, low: > 2.8 and ≤ 10.0, moderate: > 10.0 and ≤ 22.0, high: > 22.0) and Disease Activity Score in 28 joints using C‑reactive protein (DAS28-CRP; remission: < 2.6, low: ≥ 2.6 and < 3.2, moderate: ≥ 3.2 and ≤ 5.1, high: > 5.1). Dose adjustments of filgotinib and details on concomitant therapy with GCs and csDMARDs were also recorded.

Subgroup analyses were conducted in patients initiating filgotinib 100 mg versus 200 mg, patients receiving filgotinib monotherapy versus combination therapy (i.e., filgotinib with GCs/MTX) and patients with and without prior treatment with advanced therapies (i.e., bDMARDs or targeted synthetic DMARDs [tsDMARDs]).

### Statistical analyses

Data were summarized descriptively, with frequencies and percentages calculated for categorical variables, and means and standard deviations (SDs) calculated for continuous variables. For the analysis of disease activity, only patients with three consecutive measures of CDAI or DAS28-CRP (i.e., at baseline, month 3 and month 6) were included. Rates of filgotinib discontinuation, dose adjustments of filgotinib, treatment escalation with GCs and/or MTX and GC tapering were assessed using Kaplan–Meier analyses during the first 12 months of follow-up; patients were censored according to patient-specific follow-up periods. Missing data, except for incomplete dates (middle of month/year), were not imputed.

Comparisons between subgroups were performed using Chi-squared and Fisher exact tests for categorical variables, Mann–Whitney U‑tests and independent (unpaired) t‑tests for continuous variables and the log-rank test for Kaplan–Meier analyses.

## Results

### Patients and baseline characteristics

A total of 301 patients from 20 rheumatology practices across Germany were included in the study. Most patients were female (*n* = 244, 81.1%), aged < 65 years (*n* = 202, 67.1%), were positive for both RF and ACPAs (*n* = 167, 55.5%), and had no history of herpes zoster infection (*n* = 272, 90.4%; Table 1). Overall, 99 patients (32.9%) were aged ≥ 65 years and 89 (29.6%) were former/current smokers. More than one-third of patients had a disease duration of ≥ 10 years. Mean (SD) CDAI (*n* = 231) and DAS28-CRP (*n* = 230) scores (25.4 [10.9] and 4.8 [1.1], respectively) indicated that patients had moderate to severe disease activity at filgotinib initiation. The mean (SD) follow-up time was 7.9 (4.0) months.

**Table 1:** Baseline patient characteristics

	Total (*N* = 301)
*Age in years, mean (SD)*	59.2 (12.4)
*Age group, n (%)*	
< 65 years	202 (67.1)
≥ 65 to 74 years	62 (20.6)
≥ 75 years	37 (12.3)
*Female, n (%)*	244 (81.1)
*CDAI, mean (SD)*	*n* = 231 25.4 (10.9)
*DAS28-CRP, mean (SD)*	*n* = 230 4.8 (1.1)
*Disease duration, n (%)*	
*<* *1 year*	11 (3.7)
*1–5 years*	86 (28.6)
*5–10 years*	89 (29.6)
*>* *10 years*	115 (38.2)
*Serologic status*^*a*^*, n (%)*	
*RF positive only*	21 (7.0)
*ACPA positive only*	24 (8.0)
*RF and ACPA positive*	167 (55.5)
*RF and ACPA negative*	86 (28.6)
*No previous HZ infection, n (%)*	272 (90.4)
*HZ vaccination, n (%)*	
*Yes*	80 (26.6)
Unknown	85 (28.2)
*Smoking status, n (%)*	
Current smoker	43 (14.3)
Former smoker	46 (15.3)
Nonsmoker	140 (46.5)
Unknown	72 (23.9)

*ACPA* anti-cyclic citrullinated peptide antibody, *CDAI* Clinical Disease Activity Index, *DAS28-CRP* Disease Activity Score in 28 joints using C‑reactive protein, *HZ* herpes zoster, *RF* rheumatoid factor, *SD* standard deviation

^a^RF positive but ACPA unknown (*n* = 1, 0.3%), ACPA positive but RF unknown (*n* = 1, 0.3%), ACPA negative but RF unknown (*n* = 1, 0.3%)

Most patients (*n* = 255, 84.7%) received the 200 mg dose of filgotinib, initiated filgotinib as monotherapy (*n* = 252, 83.7%; of whom *n* = 32, 12.7% with GCs) and had previously received treatment with advanced DMARDs (*n* = 228, 75.7%; Table S1). Among patient subgroups, baseline characteristics were generally consistent (Table S1); more patients initiating filgotinib 100 mg were aged ≥ 65 years than those initiating 200 mg (78.3% vs 24.7%). In addition, patients who were naïve to advanced DMARDs had a shorter disease duration than patients who had previously received advanced DMARDs, with 23.3% versus 43.0% reporting a disease duration of > 10 years.

### Comorbidities

Approximately half of patients had ≥ 1 CV risk factor (*n* = 140, 46.5%), the most common of which was arterial hypertension (*n* = 103, 34.2%; Table 2). Other common comorbidities included osteoarthritis, osteoporosis/osteopenia and obesity (body mass index ≥ 30 kg/m^2^). One-fifth of patients did not report any comorbidity. Numerically, the proportion of patients with CV risk factors was higher in the 100 mg than the 200 mg subgroup, with the exception of dyslipidemia. The proportions of patients with a history of malignancy were low in both subgroups (Table S2).

**Table 2:** Baseline comorbidities

	Total (*N* = 301), *n* (%)
**CV disease**	
*Any CV risk factor*^*a*^	140 (46.5)
Arterial hypertension	103 (34.2)
Dyslipidemia	39 (13.0)
Diabetes mellitus	32 (10.6)
Cardiac arrhythmias	16 (5.3)
Coronary heart disease	13 (4.3)
Condition following myocardial or cerebral infarction	9 (3.0)
Condition following deep vein thrombosis or pulmonary embolism	8 (2.7)
Heart failure	7 (2.3)
**Metabolic syndrome**	
*Obesity (body mass index ≥* *30* *kg/m*^*2*^*)*	35 (11.6)
**Cancers**	
*Other cancers*	9 (3.0)
*Nonmelanoma skin cancer*	3 (1.0)
**Gastroenterological diseases**	
*Liver disease*	11 (3.7)
*Gastroesophageal reflux disease*	8 (2.7)
*Inflammatory bowel disease*	6 (2.0)
**Pulmonary diseases**	
*Bronchial asthma*	15 (5.0)
*Chronic obstructive pulmonary disease*	15 (5.0)
*Interstitial lung disease*	2 (0.7)
Diseases of the musculoskeletal system and connective tissue	
*Osteoarthritis (arthritis)*	91 (30.2)
*Osteoporosis/osteopenia*	65 (21.6)
*Psoriasis*	11 (3.7)
*Gout (arthritis urica)*	6 (2.0)
*Other connective tissue diseases*^*b*^	4 (1.3)
**Other comorbidities**	
*Renal insufficiency (creatinine clearance <* *60* *mL/min)*	16 (5.3)
*Anemia*	14 (4.7)
*Thyroid dysfunction*	11 (3.7)
*Vitamin D deficiency*	10 (3.3)
*Depression/anxiety/panic*	10 (3.3)
*Fibromyalgia*	5 (1.7)
*Aneurysm*	3 (1.0)
*Sigmoid diverticulitis*	3 (1.0)
*Allergies*	3 (1.0)
*Epilepsy*	2 (0.7)
**No comorbidities reported**	62 (20.6)

*CV* cardiovascular

^a^Patients may have ≥ 1 CV risk factor

^b^For example, myositis, systemic lupus erythematous, systemic sclerosis, Sjögren’s syndrome

### Prior treatments

Most patients had been previously treated with csDMARDs (*n* = 282, 93.7%) and/or GCs (*n* = 241, 80.1%; Table 3). Prior bDMARDs and tsDMARDs were received by 199 (66.1%) and 113 (37.6%) patients, respectively, and approximately one-third (29.9%) of patients had received ≥ 3 prior b/tsDMARDs. The last treatments before initiating filgotinib included csDMARDs (*n* = 120, 43.0%), bDMARDs (*n* = 97, 34.8%), GCs (*n* = 88, 31.5%), and tsDMARDs (*n* = 63, 22.6%; Fig. S1). The most common reasons for discontinuing prior treatment included primary lack of effectiveness (*n* = 144, 37.3%), adverse events (*n* = 88, 22.8%), and secondary lack of effectiveness (*n* = 86, 22.3%).

**Table 3:** Prior treatments

	Total (*N* = 301), *n* (%)
**Prior treatments**	
Glucocorticoids	241 (80.1)
csDMARDs	282 (93.7)
bDMARDs	199 (66.1)
***Tumor necrosis factor inhibitors***	
Etanercept	108 (35.9)
Adalimumab	91 (30.2)
Certolizumab pegol	45 (15.0)
Golimumab	16 (5.3)
Infliximab	16 (5.3)
***Interleukin‑6 receptor inhibitors***	
Tocilizumab	65 (21.6)
Sarilumab	23 (7.6)
Abatacept	57 (18.9)
Rituximab	18 (6.0)
*tsDMARDs*	113 (37.6)
**Number of prior b/tsDMARDs**	
0	73 (24.3)
1	87 (28.9)
2	51 (16.9)
≥ 3	90 (29.9)

*b/cs/tsDMARD* biologic/conventional synthetic/targeted synthetic disease-modifying antirheumatic drug

### Reasons for initiating filgotinib

**Fig. 1:**
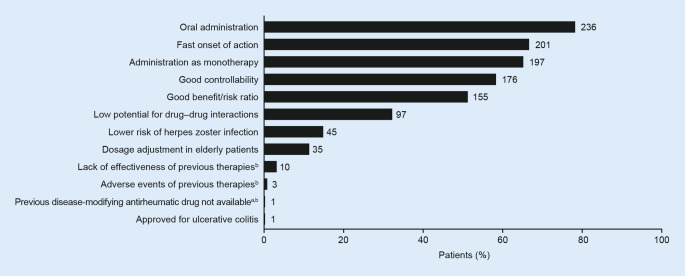
Reasons^a^ for initiating filgotinib (primary objective). Values at end of bars are numbers of patients. ^a^Multiple reasons for initiating filgotinib could be selected. ^b^Previous treatment included methotrexate, leflunomide, adalimumab, and tocilizumab

The most frequently reported reasons for initiating filgotinib included oral administration (*n* = 236, 78.4%), fast onset of action (*n* = 201, 66.8%) and administration as monotherapy (*n* = 197, 65.4%; Fig. 1). In subgroup analyses (Table S3), the potential for dosage adjustment in elderly patients was a significantly more frequent reason for initiating filgotinib 100 mg versus 200 mg. Oral administration, fast onset of action and good benefit/risk profile were more frequent reasons for initiating filgotinib as combination therapy with GCs/MTX versus as monotherapy.

### Discontinuation of filgotinib

**Fig. 2:**
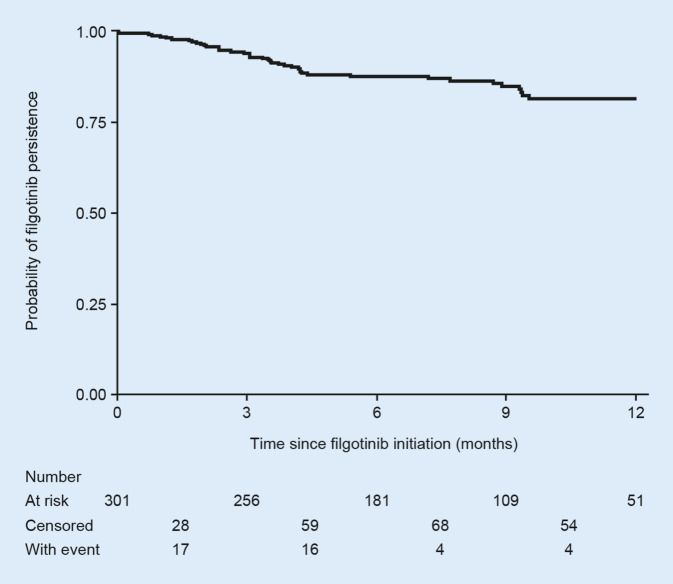
Persistence rate of filgotinib. Patients without event (e.g., those lost to follow-up) were censored at the last visit or at the last available date

After 6 months, 33 patients (12.2%) had discontinued filgotinib; 41 (18.4%) discontinued at 12 months (Fig. 2). In total, 43 (14.3%) patients discontinued filgotinib during the overall follow-up period (Table 4). The most common reason for discontinuing filgotinib was lack of effectiveness (*n* = 26, 8.6%), followed by adverse events (*n* = 12, 4.0%) and lack of adherence (*n* = 4, 1.3%). Of the 26 patients who discontinued filgotinib due to lack of effectiveness, 22 (84.6%) had previously received an advanced DMARD and 15 (57.7%) had received prior JAK inhibitors. Gastrointestinal complaints, dizziness/vertigo and infections were the most frequent adverse events leading to discontinuation (each reported for < 1% of patients).

**Table 4:** Reasons for discontinuing filgotinib

	Total (*N* = 301), *n* (%)
*Lack of effectiveness*	26 (8.6)
*Adverse events*^a^	12 (4.0)
Gastrointestinal complaints	5 (1.7)
Dizziness/vertigo	3 (1.0)
Infections	2 (0.7)
Fever	1 (0.3)
Genital inflammation	1 (0.3)
Globus pharyngeus	1 (0.3)
Polyuria	1 (0.3)
Rhinorrhea	1 (0.3)
Sweating	1 (0.3)
Urinary incontinence	1 (0.3)
*Lack of drug adherence*	4 (1.3)
*Remission*	1 (0.3)

^a^Patients may have discontinued due to > 1 adverse event

No significant differences in filgotinib discontinuation rates were observed in subgroups defined by initial filgotinib dose (100 mg vs 200 mg) or prior use of advanced DMARDs (Fig. S2). Furthermore, no differences in discontinuation rates were observed between patients treated with filgotinib as monotherapy versus combination therapy (data not shown; *p* = 0.213), although the group sizes were imbalanced. Common reasons for discontinuation were similar across patients initiating filgotinib 100 mg versus 200 mg and across patients who had previously received an advanced DMARD versus those who were advanced DMARD naïve (Table S4).

### Disease activity with filgotinib

**Fig. 3:**
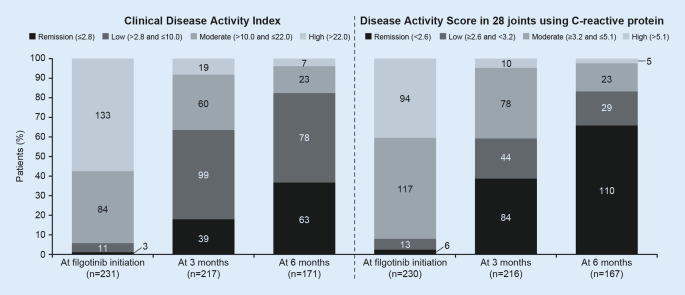
Changes in disease activity with filgotinib. Values in bar segments are numbers of patients. (Reproduced from Schultz et al. [28], with permission from © 2023 BMJ Publishing Group Ltd)

At filgotinib initiation, around 90% of patients had moderate or high disease activity based on CDAI (*n* = 217/231) or DAS28-CRP (*n* = 211/230; Fig. 3). After 6 months of filgotinib, 63/171 (36.8%) patients had achieved CDAI remission and 78/171 (45.6%) had achieved CDAI LDA. As expected, 6‑month remission rates were higher with DAS28-CRP than CDAI, with 110/167 (65.9%) and 29/167 (17.4%) patients achieving DAS28-CRP remission and LDA, respectively.

No significant differences in remission rates were observed between patients initiating filgotinib 100 mg versus 200 mg or among patients who had previously received an advanced DMARD versus those who were advanced DMARD naïve (Fig. S3). There were also no differences in remission rates between patients who initiated monotherapy versus combination therapy (data not shown; CDAI: *p* = 0.929; DAS28-CRP: *p* = 0.562).

### Concomitant treatments

Overall, 252 (83.7%) patients initiated filgotinib as monotherapy and 32 (12.7%) with GCs. The remaining 49 (16.3%) patients initiated filgotinib as combination therapy with MTX and 23 (46.9%) with GCs. Of the 55 (18.2%) patients who received concomitant GCs, 37 (67.3%) received a dose of ≥ 5 mg/day, and the mean (SD) dose of GCs was 5.4 (4.4) mg/day. Of the 49 (16.3%) patients who received concomitant MTX, the mean (SD) dose was 10.3 (3.5) mg/week in patients receiving oral treatment (*n* = 29, 59.2%) and 12.2 (4.7) mg/week in those receiving subcutaneous treatment (*n* = 20, 40.8%).

Of 54 patients who initiated filgotinib with concomitant GCs (one patient excluded due to missing values for prescription dates of concomitant GC treatment), 29 tapered or discontinued GCs within 6 months (median 5.3 months). For patients on filgotinib monotherapy, at 6 months, 13.8% (*n* = 33) had received treatment with a concomitant therapy. At 12 months, 35 (19.6%) patients on filgotinib monotherapy received treatment with a concomitant therapy (MTX, GC or a combination of both).

### Filgotinib dose adjustment

During the follow-up period, 20 (6.6%) patients had their dose of filgotinib adjusted (reasons were not documented); a dose increase from 100 to 200 mg occurred in 7/46 (15.2%) patients, while a dose decrease from 200 to 100 mg occurred in 13/255 (5.1%) patients.

## Discussion

Filgotinib has shown efficacy and safety in patients with RA in several RCTs [6–9]. However, while RCTs are considered the ‘gold standard’ for assessing a drug’s efficacy, their stringent inclusion and exclusion criteria can limit their external validity [10] as patients with multiple comorbidities or specific previous treatments are often excluded, and elderly patients may be underrepresented. For example, only 19% of patients were ≥ 65 years old in the pooled filgotinib phase 2 and 3 trials [11]. Observational studies can complement RCTs by assessing the effects of a drug in real-world populations [10, 12]. This multicenter, retrospective medical chart review was performed to gather real-world insights into the use of filgotinib in patients with RA in rheumatology practices in Germany. In this real-world study spanning from the day filgotinib became available (October 15, 2020) until completion of data collection on May 2, 2022, comorbidities and CV risk factors were common, and around one-third of patients were ≥ 65 years old. One-third of patients had arterial hypertension, slightly fewer than reported in a previous study using data from the German RABBIT registry, in which 47.7% of 713 patients with RA treated with JAK inhibitors (tofacitinib, baricitinib, and upadacitinib) had hypertension [13]. In contrast, the recent ORAL Surveillance study reported a higher risk of major adverse CV events and malignancies in patients aged 50 years or older with ≥ 1 additional CV risk factor receiving tofacitinib compared with tumor necrosis factor (TNF) inhibitors [14].

We observed over half of patients with RA were seropositive for both RF and ACPA, and that 28.6% were autoantibody-negative in our study population, which is broadly in line with existing epidemiological data which suggests a frequency of 20–30% [15]. No analysis of treatment response in the different seropositive and seronegative subgroups was performed, and the robustness of such subgroup analyses would be limited. To date, no study has been specifically designed to compare these subgroups, so this remains a topic of interest.

The most frequently cited reason for initiating filgotinib was oral route of administration. This is in keeping with prior studies, which have shown that route of administration was a key factor for patients with RA, and that the majority prefer the oral route. In a previous choice-based survey of 380 patients with RA, route of administration was ranked the most important medication attribute, and 56.4% of patients preferred the oral route over intravenous or subcutaneous administration [16]. In addition, a discrete-choice experiment with 1588 patients with RA in Germany found that oral administration was the most desired characteristic of an RA medication [17] and that patients preferred medications that did not require concomitant MTX.

Several biologics (i.e., infliximab, abatacept, and rituximab) are only approved in combination with MTX for RA [18–20]. Moreover, although TNF inhibitors such as adalimumab and etanercept can be given as monotherapy, greater efficacy has been observed when these agents are combined with MTX [21, 22]. Filgotinib is frequently used as monotherapy, as demonstrated in the ongoing European real-world FILOSOPHY (NCT04871919) study of filgotinib in patients with RA, where filgotinib monotherapy was received by 65.7% of 242 patients in the German cohort [23] and by 52.9% of 480 patients overall [24]. Similarly, in the present study, around 80% of patients initiated filgotinib as monotherapy, and we found that the option to use filgotinib as monotherapy was a frequently cited reason for initiating treatment. This is consistent with real-world studies of other JAK inhibitors, in which baricitinib and tofacitinib were often used as monotherapy [25, 26]. However, the frequency of monotherapy use was higher in this study (83.7%) than in the tofacitinib and baricitinib studies (53.1 and 43.4%, respectively). This may reflect the differences in healthcare systems in the US and Spain compared to Germany.

While the recommended dose of filgotinib is 200 mg once daily (QD), at the time of study initiation, a starting dose of 100 mg QD was recommended in elderly patients (≥ 75 years old) and patients with moderate or severe renal impairment. Following a recent label change for all JAK inhibitors, filgotinib 100 mg QD (with the possibility to escalate to 200 mg QD) is currently recommended in elderly patients (≥ 65 years old); patients with moderate or severe renal impairment; and patients at risk of major adverse CV events (such as current or past long-term smokers), venous thromboembolism or malignancy, if no suitable treatment alternatives are available [5]. Around 15% of patients initiated filgotinib 100 mg in this study, and they achieved a similar response compared to the 200 mg dose; this is in contrast to existing studies [27] and may be attributed to the study design and selection bias. As might be expected, a higher proportion of patients initiating filgotinib 100 mg in this study were aged ≥ 65 years than those initiating the 200 mg dose, and proportionally more had renal insufficiency and ≥ 1 CV risk factor. The potential for dosage adjustment in elderly patients was reported as a reason for starting filgotinib in around half of patients who initiated at a dose of 100 mg. However, dose increases were relatively rare during this study (occurring in only 15.2% of patients starting on filgotinib 100 mg).

Favorable effectiveness results were observed in this study, with > 50% and > 80% of patients achieving CDAI or DAS28-CRP remission or LDA within 3 and 6 months of treatment, respectively, and a relatively low rate of discontinuation. This is notable, given the treatment-refractory nature of the population, with almost one-third having received ≥ 3 prior b/tsDMARDs. This suggests that filgotinib can be effective in patients for whom multiple prior advanced therapies have failed, although the follow-up time was limited (mean 7.9 months), and longer-term data from larger, prospective cohorts are needed to confirm this. In the FINCH RCTs, CDAI and DAS28-CRP remission rates for filgotinib 200 mg were 12% and 22–34% at week 12 [6, 7], and 21 and 42% at week 24 [9], respectively. CDAI and DAS28-CRP LDA was achieved by 46% and 41–50% of patients receiving filgotinib 200 mg at week 12 [6, 7].

A limitation of this study is that all patients were recruited in Germany, which may reduce the generalizability of the findings to patients in other countries in and outside Europe, but better reflects the treatment reality in Germany compared to a study conducted in several countries. Furthermore, disease activity analyses were not available for all patients and lack a comparator (placebo/active), meaning that results should be interpreted with caution. The initiation of GCs and csDMARDs during the follow-up period may also have impacted the effectiveness findings. Additional limitations include the retrospective design of the study, small number of study sites, possible patient selection bias favoring positive outcomes and that the study was designed prior to the recent label change for JAK inhibitors, precluding conclusions on safety points such as infections and CV and embolism risk.

## Conclusions

These results provide important information about the use of filgotinib in patients with rheumatoid arthritis (RA) in a real-world context in Germany from the day it became available (October 15, 2020) until completion of data collection on May 2, 2022. Filgotinib was used predominantly as monotherapy, which was one of the main reasons given for initiation, along with fast onset of action (as demonstrated in the FILOSOPHY study) and oral route of administration. The 100 mg dose was used more frequently than the 200 mg dose in elderly patients and those with renal impairment and cardiovascular (CV) risk factors. Effectiveness and tolerability appeared favorable; the ongoing FILOSOPHY study will provide longer-term data from follow-up of patients with RA receiving filgotinib in a real-world setting.

## Supplementary Information

Supplementary Appendix (Tab. S1–S4, Fig. S1–S3)

## Data Availability

Anonymized individual patient data will be shared upon request for research purposes dependent upon the nature of the request, the merit of the proposed research and the availability of the data and its intended use. The data sharing policy for Galapagos NV can be found at https://www.clinicaltrials-glpg.com/us/en/data-transparency.html.

## Declarations

For this article no studies with human participants or animals were performed by any of the authors. All studies mentioned were in accordance with the ethical standards indicated in each case.
