# Supplementary material for: Real-world experience with filgotinib for rheumatoid arthritis in Germany: A retrospective chart review
Source: Z Rheumatol. 2024 Apr 30;84(1):39–47. doi: 10.1007/s00393-024-01506-x (PMC11779788; doi:10.1007/s00393-024-01506-x)
Supplement: Supplementary file 1 — Supplementary Appendix (Tab. S1–S4, Fig. S1–S3) [file 393_2024_1506_MOESM1_ESM.pdf]

## Supplementary Appendix

**Table S1.** Baseline patient characteristics by subgroup

| | FIL200 (n=255) | FIL100 (n=46) | FIL monotherapy (n=220) | FIL + GCs/MTX (n=81) | Prior advanced DMARD (n=228) | No prior advanced DMARD (n=73) |
|---|---|---|---|---|---|---|
| Age, mean (SD), years | **57.1 (11.3)**[a] | **70.6 (12.2)**[a] | 59.7 (12.7) | 57.8 (11.7) | 58.7 (12.2) | 60.7 (13.0) |
| Age group, n (%) | | | | | | |
| <65 years | 192 (75.3) | 10 (21.7) | 140 (63.6) | 62 (76.5) | 159 (69.7) | 43 (58.9) |
| ≥65 to 74 years | 50 (19.6) | 12 (26.1) | 52 (23.7) | 10 (12.3) | 41 (18.0) | 21 (28.8) |
| ≥75 years | 13 (5.1) | 24 (52.2) | 28 (12.7) | 9 (11.1) | 28 (12.3) | 9 (12.3) |
| Female, n (%) | 203 (79.6) | 41 (89.1) | 179 (81.4) | 65 (80.3) | 183 (80.3) | 61 (83.6) |

| | | | | | | |
|---|---|---|---|---|---|---|
| CDAI, mean (SD) | n=195<br>22.5 (12.8) | n=36<br>25.9 (10.5) | n=169<br>25.9 (11.2) | n=62<br>24.0 (10.2) | n=170<br>24.9 (11.0) | n=61<br>26.8 (10.7) |
| DAS28-CRP, mean (SD) | n=36<br>4.9 (1.4) | n=178<br>5.1 (1.2) | n=162<br>5.2 (1.2) | n=52<br>4.9 (1.2) | n=155<br>5.0 (1.3) | n=59<br>5.3 (1.1) |
| Disease duration, n (%) | | | | | | |
| <1 year | 10 (3.9) | 1 (2.2) | 10 (4.5) | 1 (1.2) | **0**[a] | **11 (15.1)**[a] |
| 1–5 years | 76 (29.8) | 10 (21.7) | 56 (25.5) | 30 (37.0) | **58 (25.4)**[a] | **28 (38.4)**[a] |
| 5–10 years | 79 (31.0) | 10 (21.7) | 65 (29.6) | 24 (29.6) | **72 (31.6)**[a] | **17 (23.3)**[a] |
| >10 years | 90 (35.3) | 25 (54.4) | 89 (40.5) | 26 (32.1) | **98 (43.0)**[a] | **17 (23.3)**[a] |
| Serologic status,[b] n (%) | | | | | | |
| RF positive only | 17 (6.7) | 4 (8.7) | 16 (7.3) | 5 (6.2) | 15 (6.6) | 6 (8.2) |
| ACPA positive only | 21 (8.2) | 3 (6.5) | 18 (8.2) | 6 (7.4) | 17 (7.5) | 7 (9.6) |

| | | | | | | |
|---|---|---|---|---|---|---|
| RF and ACPA positive | 143 (56.1) | 24 (52.2) | 118 (53.6) | 49 (60.5) | 128 (56.1) | 39 (53.4) |
| RF and ACPA negative | 71 (27.8) | 15 (32.6) | 66 (30.0) | 20 (24.7) | 66 (29.0) | 20 (27.4) |
| No previous HZ infection, n (%) | 234 (91.8) | 38 (82.6) | 200 (90.9) | 72 (88.9) | 202 (88.6) | 70 (95.9) |
| HZ vaccination, n (%) | | | | | | |
| Yes | 67 (26.3) | 13 (28.3) | 63 (28.6) | 17 (21.0) | 56 (24.6) | 24 (32.9) |
| Unknown | 70 (27.5) | 15 (32.6) | 59 (26.8) | 26 (32.1) | 71 (31.1) | 14 (19.2) |
| Smoking status, n (%) | | | | | | |
| Current smoker | 42 (16.5) | 1 (2.2) | 27 (12.3) | 16 (19.8) | 32 (14.0) | 11 (15.1) |
| Former smoker | 39 (15.3) | 7 (15.2) | 31 (14.1) | 15 (18.5) | 36 (15.8) | 10 (13.7) |
| Nonsmoker | 114 (44.7) | 26 (56.5) | 107 (48.6) | 33 (40.7) | 106 (46.5) | 34 (46.6) |

| | | | | | | |
|---|---|---|---|---|---|---|
| Unknown | 60 (23.5) | 12 (26.1) | 55 (25.0) | 17 (21.0) | 54 (23.7) | 18 (24.7) |
| Follow-up period, mean (SD), months | **8.2 (4.0)[a]** | **6.2 (3.3)[a]** | 7.8 (4.2) | 8.3 (3.3) | 7.8 (3.9) | 8.4 (4.1) |

[a]p<0.05. For continuous variables, nonparametric Mann–Whitney U-tests and independent (unpaired) t-tests were considered. Categorical variables were compared using Chi-squared and Fisher exact tests

[b]FIL200: RF positive but ACPA unknown (n=1, 0.4%), ACPA positive but RF unknown (n=1, 0.4%), ACPA negative but RF unknown (n=1, 0.4%). FIL monotherapy: ACPA positive but RF unknown (n=1, 0.5%), ACPA negative but RF unknown (n=1, 0.5%). FIL + GCs/MTX: RF positive but ACPA unknown (n=1, 1.2%). Prior advanced DMARD: ACPA positive but RF unknown (n=1, 0.4%), ACPA negative but RF unknown (n=1, 0.4%). No prior advanced DMARD: ACPA positive but RF unknown (n=1, 1.4%)

ACPA, anti-cyclic citrullinated peptide antibody; CDAI, Clinical Disease Activity Index; DAS28-CRP, Disease Activity Score in 28 joints using C-reactive protein; DMARD, disease-modifying antirheumatic drug; FIL(100/200), filgotinib (100/200 mg); GC, glucocorticoid; HZ, herpes zoster; MTX, methotrexate; RF, rheumatoid factor; SD, standard deviation

**Table S2.** Baseline comorbidities by subgroup

| n (%) | FIL200 (n=255) | FIL100 (n=46) | FIL monotherapy (n=220) | FIL + GCs/MTX (n=81) | Prior advanced DMARD (n=228) | No prior advanced DMARD (n=73) |
|---|---|---|---|---|---|---|
| CV disease | | | | | | |
| Any CV risk factor[a] | 113 (44.3) | 27 (58.7) | 101 (45.9) | 39 (48.1) | 110 (48.2) | 30 (41.1) |
| Arterial hypertension | 79 (31.0) | 24 (52.2) | 31 (14.1) | 72 (88.9) | 83 (36.4) | 20 (27.4) |
| Dyslipidemia | 37 (14.5) | 2 (4.3) | 4 (1.8) | 35 (43.2) | 28 (12.3) | 11 (15.1) |
| Diabetes mellitus | 22 (8.6) | 10 (21.7) | 7 (3.2) | 25 (30.9) | 28 (12.3) | 4 (5.5) |
| Cardiac arrhythmias | 11 (4.3) | 5 (10.9) | 6 (2.7) | 10 (12.3) | 11 (4.8) | 5 (6.8) |
| Coronary heart disease | 9 (3.5) | 4 (8.7) | 3 (1.4) | 10 (12.3) | 12 (5.3) | 1 (1.4) |
| Condition following myocardial or cerebral infarction | 5 (2.0) | 4 (8.7) | 4 (1.8) | 5 (6.2) | 9 (3.9) | 0 |
| Condition following deep vein thrombosis or pulmonary embolism | 6 (2.4) | 2 (4.3) | 4 (1.8) | 4 (4.9) | 8 (3.5) | 0 |
| Heart failure | 4 (1.6) | 3 (6.5) | 4 (1.8) | 3 (3.7) | 7 (3.1) | 0 |
| Metabolic syndrome | | | | | | |

| | | | | | | |
|---|---|---|---|---|---|---|
| Obesity (body mass index ≥30 kg/m$^2$) | 34 (13.3) | 1 (2.2) | 12 (5.5) | 23 (28.4) | 23 (10.1) | 12 (16.4) |
| Cancers | | | | | | |
| Other cancers | 6 (2.4) | 3 (6.5) | 1 (0.5) | 8 (9.9) | 8 (13.6) | 1 (1.4) |
| Nonmelanoma skin cancer | 2 (0.8) | 1 (2.2) | 2 (0.9) | 1 (1.2) | 2 (0.9) | 1 (1.4) |
| Gastroenterological diseases | | | | | | |
| Liver disease | 10 (3.9) | 1 (2.2) | 2 (0.9) | 9 (11.1) | 10 (4.4) | 1 (1.4) |
| Gastroesophageal reflux disease | 8 (3.1) | 0 | 4 (1.8) | 4 (4.9) | 6 (2.6) | 2 (2.7) |
| Inflammatory bowel disease | 6 (2.4) | 0 | 0 | 6 (7.4) | 5 (2.2) | 1 (1.4) |
| Pulmonary diseases | | | | | | |
| Bronchial asthma | 12 (4.7) | 3 (6.5) | 0 | 15 (18.5) | 14 (6.1) | 1 (1.4) |
| Chronic obstructive pulmonary disease | 12 (4.7) | 3 (6.5) | 6 (2.7) | 9 (11.1) | 12 (5.3) | 3 (4.1) |
| Interstitial lung disease | 2 (0.8) | 0 | 1 (0.5) | 1 (1.2) | 1 (0.4) | 1 (1.4) |
| Diseases of the musculoskeletal system and connective tissue | | | | | | |
| Osteoarthritis (arthritis) | 73 (28.6) | 18 (39.1) | 23 (10.5) | 68 (84.0) | 70 (30.7) | 21 (28.8) |
| Osteoporosis/osteopenia | 46 (18.0) | 19 (41.3) | 17 (7.7) | 48 (59.3) | 50 (21.9) | 15 (20.5) |

| | | | | | | |
|---|---|---|---|---|---|---|
| Psoriasis | 8 (3.1) | 3 (6.5) | 2 (0.9) | 9 (11.1) | 11 (4.8) | 0 |
| Gout (arthritis urica) | 5 (2.0) | 1 (2.2) | 2 (0.9) | 4 (4.9) | 5 (2.2) | 1 (1.4) |
| Other connective tissue diseases[b] | 4 (1.6) | 0 | 2 (0.9) | 2 (2.5) | 4 (1.8) | 0 |
| Other comorbidities | | | | | | |
| Renal insufficiency (creatinine clearance <60 mL/min) | 6 (2.4) | 10 (21.7) | 2 (0.9) | 14 (17.3) | 14 (6.1) | 2 (2.7) |
| Anemia | 10 (3.9) | 4 (8.7) | 3 (1.4) | 11 (13.6) | 10 (4.4) | 4 (5.5) |
| Thyroid dysfunction | 9 (3.5) | 2 (4.3) | 6 (2.7) | 5 (6.2) | 9 (3.9) | 2 (2.7) |
| Vitamin D deficiency | 9 (3.5) | 1 (2.2) | 3 (1.4) | 7 (8.6) | 9 (3.9) | 1 (1.4) |
| Depression / anxiety / panic | 7 (2.7) | 3 (6.5) | 3 (1.4) | 7 (8.6) | 8 (3.5) | 2 (2.7) |
| Fibromyalgia | 4 (1.6) | 1 (2.2) | 4 (1.8) | 1 (1.2) | 5 (2.2) | 0 (0) |
| Aneurysm | 3 (1.2) | 0 (0) | 1 (0.5) | 2 (2.5) | 3 (1.3) | 0 (0) |
| Sigmoid diverticulitis | 3 (1.2) | 0 (0) | 0 (0) | 3 (3.7) | 2 (0.9) | 1 (1.4) |
| Allergies | 1 (0.4) | 2 (4.3) | 0 (0) | 3 (3.7) | 3 (1.3) | 0 (0) |
| Epilepsy | 2 (0.8) | 0 (0) | 0 (0) | 2 (2.5) | 2 (0.9) | 0 (0) |
| No comorbidities reported | 57 (22.4) | 5 (10.9) | 15 (6.8) | 47 (58.0) | 45 (19.7) | 17 (23.3) |

[a]Patients may have ≥1 CV risk factor

[b]For example, myositis, systemic lupus erythematous, systemic sclerosis, Sjögren's syndrome

CV, cardiovascular; DMARD, disease-modifying antirheumatic drug; FIL(100/200), filgotinib (100/200 mg); GC, glucocorticoid; MTX, methotrexate

**Table S3.** Reasons for initiating filgotinib by subgroup (primary objective)

| n (%) | FIL200 (n=255) | FIL100 (n=46) | FIL monotherapy (n=220) | FIL+ GCs/MTX (n=81) | Prior advanced DMARD (n=228) | No prior advanced DMARD (n=73) |
|---|---|---|---|---|---|---|
| Oral administration | 197 (77.3) | 39 (84.8) | **164 (74.5)**[a] | **72 (89.0)**[a] | 176 (77.2) | 60 (82.2) |
| Fast onset of action | 172 (67.5) | 29 (63.0) | **139 (63.2)**[a] | **62 (76.5)**[a] | 154 (67.5) | 47 (64.4) |
| Administration as monotherapy | 162 (63.5) | 35 (76.1) | **155 (70.5)**[a] | **42 (51.9)**[a] | 153 (67.1) | 44 (60.3) |
| Good controllability | 144 (56.5) | 32 (69.6) | 130 (59.1) | 46 (56.8) | 129 (56.6) | 47 (64.4) |
| Good benefit/risk ratio | 127 (49.8) | 28 (60.9) | **101 (46.0)**[a] | **51 (63.0)**[a] | 115 (50.4) | 37 (50.7) |
| Low potential for drug–drug interactions | 81 (31.8) | 16 (34.8) | 75 (34.1) | 22 (27.2) | 75 (32.9) | 22 (30.1) |
| Lower risk of herpes zoster infection | 38 (14.9) | 7 (15.2) | 28 (12.7) | 17 (21.0) | 30 (13.2) | 15 (20.5) |

| | | | | | | |
|---|---|---|---|---|---|---|
| Dosage adjustment in elderly patients | **13 (5.1)**[a] | **22 (47.8)**[a] | 26 (11.8) | 9 (11.1) | 25 (11.0) | 10 (13.7) |
| Lack of effectiveness of previous therapies | 10 (3.9) | NA | 5 (2.3) | 5 (6.2) | 10 (4.4) | 0 |
| Adverse events of previous therapies | 2 (0.8) | 1 (2.2) | 3 (1.4) | NA | 3 (1.3) | NA |
| Previous DMARD not available[b] | 1 (0.4) | NA | 1 (0.5) | NA | 1 (0.4) | NA |
| Approved for ulcerative colitis | 1 (0.4) | NA | 1 (0.5) | NA | 1 (0.4) | NA |

[a]$p<0.05$. Categorical variables were compared using Chi-squared and Fisher exact tests

[b]Previous treatment included MTX, leflunomide, adalimumab and tocilizumab

DMARD, disease-modifying antirheumatic drug; FIL(100/200), filgotinib (100/200 mg); GC, glucocorticoid; MTX, methotrexate; NA, not available

**Table S4.** Reasons for discontinuing filgotinib by subgroup

| n (%) | FIL200 (n=255) | FIL100 (n=46) | Prior advanced DMARD (n=228) | No prior advanced DMARD (n=73) |
|---|---|---|---|---|
| Lack of effectiveness | 22 (8.6) | 4 (8.7) | 22 (9.6) | 4 (5.5) |
| Adverse events[a] | 8 (3.1) | 4 (8.7) | 8 (3.5) | 4 (5.5) |
| Gastrointestinal complaints | 3 (37.5) | 2 (50.0) | 3 (42.9) | 2 (50.0) |
| Dizziness / vertigo | 2 (25.0) | 1 (25.0) | 3 (42.9) | 0 (0) |
| Infections | 1 (12.5) | 1 (25.0) | 1 (14.3) | 1 (25.0) |
| Fever | 1 (12.5) | 0 (0) | 0 (0) | 1 (25.0) |
| Genital inflammation | 1 (12.5) | 0 (0) | 1 (14.3) | 0 (0) |
| Globus pharyngeus | 1 (12.5) | 0 (0) | 1 (14.3) | 0 (0) |
| Polyuria | 1 (12.5) | 0 (0) | 1 (14.3) | 0 (0) |
| Rhinorrhea | 1 (12.5) | 0 (0) | 1 (14.3) | 0 (0) |
| Sweating | 1 (12.5) | 0 (0) | 1 (14.3) | 0 (0) |
| Urinary incontinence | 0 (0) | 1 (25.0) | 1 (14.3) | 0 (0) |
| Lack of drug adherence | 4 (1.6) | 0 | 4 (1.8) | 0 |

| | | | | |
|---|---|---|---|---|
| Remission | 1 (0.4) | 0 | 0 | 1 (1.4) |

[a]Patients may have discontinued due to >1 adverse event

DMARD, disease-modifying antirheumatic drug; FIL(100/200), filgotinib (100/200 mg)

**Fig. S1** Last treatment before initiating filgotinib

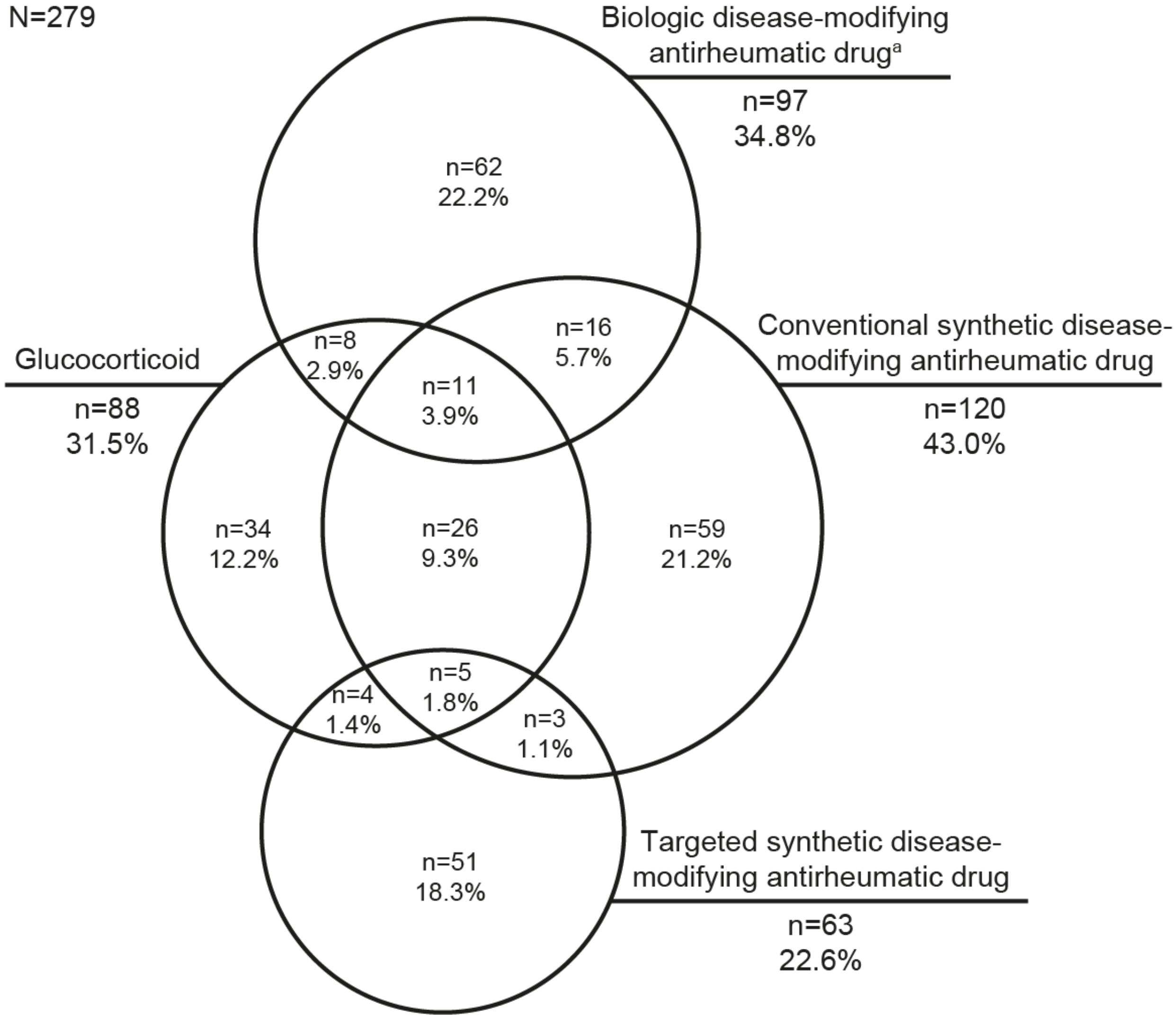


[a]One patient with ulcerative colitis was treated with ustekinumab before starting filgotinib

**Fig. S2** Persistence rates of filgotinib by subgroup: initiating filgotinib 200 versus 100 mg/day **(a)** and prior versus no prior advanced disease-modifying antirheumatic drug **(b)**

**a**

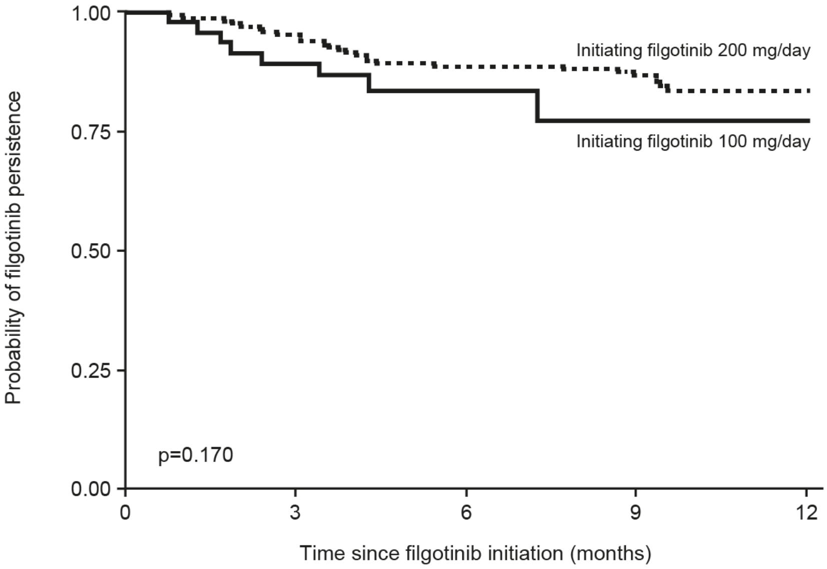


Number

| | | 0 | | 3 | | 6 | | 9 | | 12 |
|---|---|---|---|---|---|---|---|---|---|---|
| 100 mg/day | At risk | 46 | | 36 | | 18 | | 7 | | 4 |
| | With event | | 5 | | 2 | | 1 | | 0 | |
| 200 mg/day | At risk | 255 | | 220 | | 163 | | 102 | | 47 |
| | With event | | 12 | | 14 | | 3 | | 4 | |

**b**

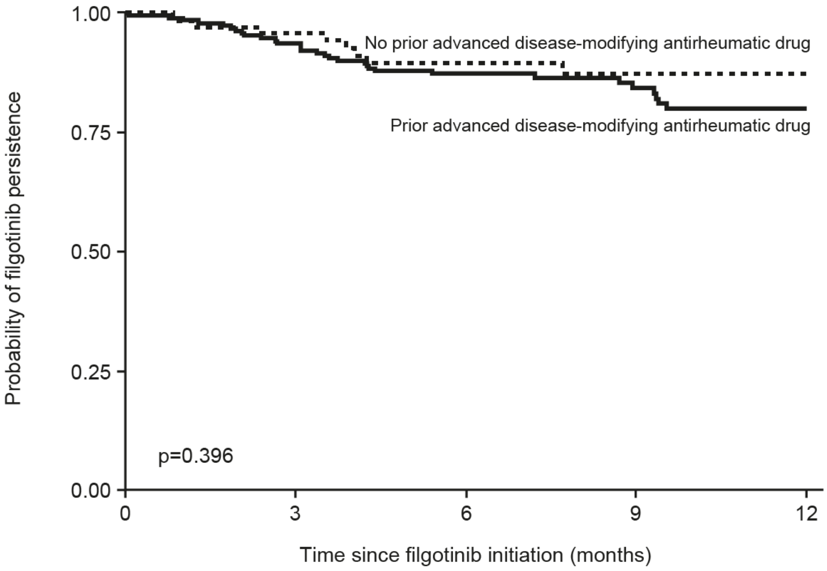


Number

| | | 0 | | 3 | | 6 | | 9 | | 12 |
|---|---|---|---|---|---|---|---|---|---|---|
| Prior | At risk | 228 | | 193 | | 133 | | 81 | | 37 |
| | With event | | 14 | | 12 | | 3 | | 4 | |
| No prior | At risk | 73 | | 63 | | 48 | | 28 | | 14 |
| | With event | | 3 | | 4 | | 1 | | 0 | |

Patients without event (e.g., those lost to follow-up) were censored at the last visit or at the last available date. Discontinuation rates were compared using the log-rank test

**Fig. S3** Clinical Disease Activity Index **(a)** and Disease Activity Score in 28 joints using C-reactive protein **(b)** ≥low (i.e., low, moderate, or high) disease activity rates with filgotinib by subgroup

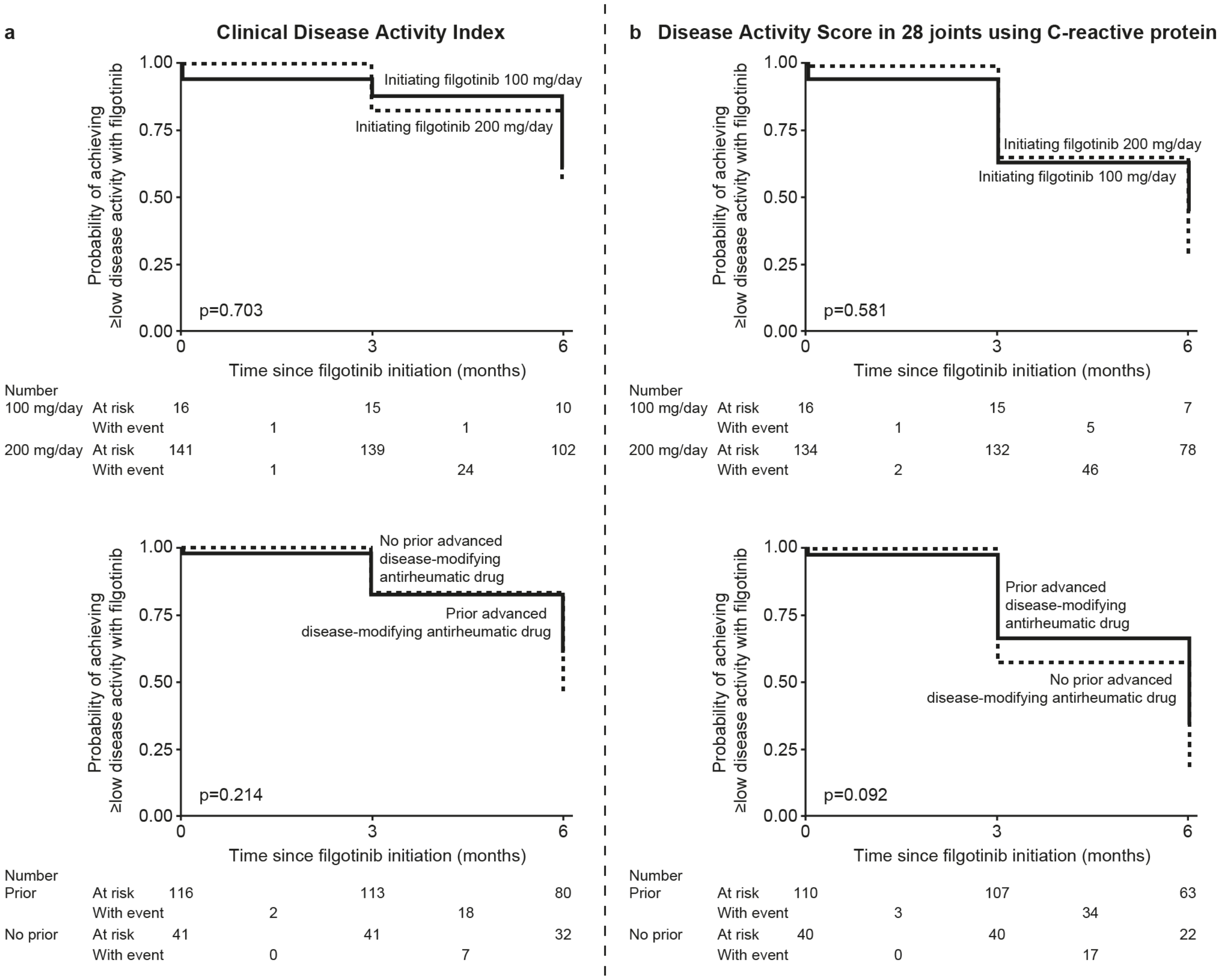


Patients with three consecutive measures of Clinical Disease Activity Index or Disease Activity Score in 28 joints using C-reactive protein, i.e., at baseline, 3 and 6 months, were included. Patients without event (e.g., those lost to follow-up) were censored at the last visit or at the last available date. Remission rates were compared using the log-rank test
